# Childhood environment influences epigenetic age and methylation concordance of a CpG clock locus in British-Bangladeshi migrants

**DOI:** 10.1080/15592294.2022.2153511

**Published:** 2022-12-10

**Authors:** Reinhard Stöger, Minseung Choi, Khurshida Begum, Gregory Leeman, Richard D. Emes, Philippa Melamed, Gillian R. Bentley

**Affiliations:** a School of Biosciences, University of Nottingham, Nottingham, UK; b School of Medicine, Stanford University, Stanford, CA, USA; c Department of Anthropology, Durham University, Durham, UK; d School of Veterinary Medicine and Science, University of Nottingham, Nottingham, UK; e Advanced Data Analysis Centre, University of Nottingham, Nottingham, UK; f Faculty of Biology, Technion-Israel Institute of Technology, Haifa, Israel; g Wolfson Research Institute for Health and Wellbeing, Durham University, Durham, UK

**Keywords:** Childhood environment, migrants, AgeAccel, methylation concordance, epigenetic clock, Bangladesh, UK

## Abstract

Migration from one location to another often comes with a change in environmental conditions. Here, we analysed features of DNA methylation in young, adult British-Bangladeshi women who experienced different environments during their childhoods: *a)* migrants, who grew up in Bangladesh with exposure to comparatively higher pathogen loads and poorer health care, and *b)* second-generation British-Bangladeshis, born to Bangladeshi parents, who grew up in the UK. We used buccal DNA to estimate DNA methylation-based age (DNAm age) from 14 migrants and 11 second-generation migrants, aged 18–35 years. ‘AgeAccel,’ a measure of DNAm age, independent of chronological age, showed that the group of women who spent their childhood in Bangladesh had higher AgeAccel (P = 0.028), compared to their UK peers. Since epigenetic clocks have been proposed to be associated with maintenance processes of epigenetic systems, we evaluated the preference for concordant DNA methylation at the luteinizing hormone/choriogonadotropin receptor (*LHCGR/LHR*) locus, which harbours one of the CpGs contributing to Horvath’s epigenetic clock. Measurements on both strands of individual, double-stranded DNA molecules indicate higher stability of DNA methylation states at this *LHCGR/LHR* locus in samples of women who grew up in Bangladesh. Together, our two independent analytical approaches imply that childhood environments may induce subtle changes that are detectable long after exposure occurred, which might reflect altered activity of the epigenetic maintenance system or a difference in the proportion of cell types in buccal tissue. This exploratory work supports our earlier findings that adverse childhood environments lead to phenotypic life history trade-offs.

## Background

Biological ageing, the waning of physiological functions over time is inherent to eukaryotes, including humans. Individuals age at different rates and this affects their health and life expectancy [1]. Twin studies suggest a moderate genetic involvement of about 25% in human lifespan variation [2–4]; an influence that becomes apparent mainly in later life past the age of 60 [2]. Stochastic processes may also cause heterogeneity in the rate at which people age [5], but environmental factors appear to have the strongest impact on human life expectancy, as shown by census data and population studies [6]. During the last seven decades, the global average life expectancy increased by about 22 years for both men (70.5 years) and women (75.6 years) [7]. The remarkable increase in lifespan is attributed to advances in hygiene, nutrition, and health care which, in concert, have significantly reduced child mortality [8]. Yet, substantial health disparities among populations persist, many of which are linked to specific geographic influences [9] that impact on life history traits, disability adjusted life years, and lifespan [10,11]. Investigators are now using molecular biomarkers, such as telomere length and epigenetic age, to gain a better understanding of the impact of environmental factors on morbidity and mortality [1,12,13].

Environmental factors influence cell‐type composition of tissues [14] and chromatin states within cells [15]. Using epigenetic information, cells have the capacity to retain some memory of past developmental and environmental conditions [16]. Methylation levels of discrete CpG sites have been used to develop remarkably accurate estimators of chronological age. Such ‘epigenetic clocks’ have been proposed to link developmental and maintenance processes with biological ageing [17–19]. Biological ageing varies across individuals, at times resulting in a mismatch between chronological and biological age [20].

Environmental conditions experienced during early life influence individuals in ways, which may affect their health during adulthood [21]. The developing body may invest limited resources towards physiological demands and biological fitness traits that enable survival during early life at the expense of other characteristics in later life [22]. Such life history ‘trade-offs’ and interactions of fitness traits include growth patterns, timing of puberty, immune function and, in adulthood, characteristics such as age at menopause and lifespan [23–26].

Our earlier work has already identified consistent associations between childhood environmental conditions and the adult phenotype [27–29]. Bangladeshi women who migrated as adults (defined as >16 years old) to London, had lower levels of salivary progesterone, a later age at puberty and earlier ages at menopause when compared to British-Bangladeshi women who moved as children to the UK and second-generation women born in London to first-generation Bangladeshi immigrants [28–31]. Age at adrenarche was also earlier among recent child migrants to the UK [31]. Geographically and culturally, the British-Bangladeshis in these studies have a comparable background and ancestry. They are all ethnic Bengalis, are mostly Muslim, and originate from a relatively affluent middle-class population living in Sylhet District in the northeast of Bangladesh [32]. Once in the UK, they traditionally settled in ethnic enclaves in east London and other neighbourhoods [33,34] but this pattern is now changing as people move out to further suburbs [35]. A childhood spent in Bangladesh is generally associated with a shortened reproductive lifespan [36] relative to women with Bangladeshi ancestry whose childhood was spent in London [28]. A possible environmental factor that distinguishes between the two childhood locations is exposure to higher and recurrent infectious disease loads in Bangladesh [36–38]. Indeed, by mimicking early-life immune challenges in a mouse model, we have replicated some of the distinct reproductive phenotypes characteristic of women with a childhood in Bangladesh, including a delayed onset of puberty and lower ovarian reserve [28,36,39].

In the current study, we explored *i)*the possible association between chronological age, biological ageing and childhood location, as well as *ii)* the stability of the epigenetic maintenance system at one location within the genome in Bangladeshi women, aged between 18 and 35 years. The women in this study lived within the same ethnic community in London but were divided into two groups: *a*) those with a childhood spent in Sylhet, who immigrated to the UK when they were >16 years of age, and *b*) those with a childhood spent in the UK (that is, born in the UK or immigrated < 2 years of age). By profiling buccal cell DNA on the Illumina 850 MethylationEpic array platform, we previously identified genome-wide, altered DNA methylation levels between the two groups of London-based Bangladeshi women [39]. Here, we used these methylation data to establish epigenetic age predictions, with three different epigenetic clocks: ‘Horvath’s multi-tissue’ clock, a ‘skin & blood’ clock, and a ‘3-CpG-swab’ model [40–42]. The tick rate of Horvath’s epigenetic clock is thought to reflect the rate at which work is undertaken to maintain epigenetic stability within a cell [17,41]. It is possible to infer epigenetic stability with a new metric, the Ratio of Concordance Preference (RCP), by analysing methylation data derived from individual, double-stranded DNA molecules [43]. We generated such data from the *LHCGR/LHR* locus to probe the efficiency of maintaining the DNA methylation state, perhaps capturing a minuscule aspect of what epigenetic clocks may register [17,18].

## Results and discussion

### Accelerated DNAm age measured with Horvath’s epigenetic clock

**Figure 1.**
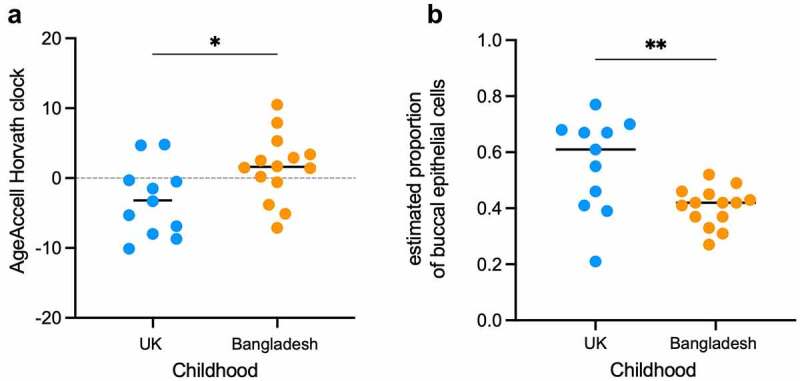
Differences in pace of epigenetic ageing and buccal cell composition.

We found that the correlation between chronological age and DNAm age did not differ significantly between women who grew up in the UK (‘UK’ group; n = 11) and women who grew up in Bangladesh (‘Bangladesh’ group; n = 14). That is, chronological age affected DNAm age in similar ways in both groups (Additional File: Figure S1 & Table 1). However, regression analysis indicated that the y-intercepts of the UK and Bangladeshi groups did differ significantly (P = 0.009) which suggested divergent DNAm age predictions between the two cohorts (Additional File: Figure S1 & Table 1). Potential differences in epigenetic age estimates and chronological age, including accelerated epigenetic ageing, are best measured by comparing the distribution of residuals obtained from a linear model, where DNAm age is regressed on chronological age (AgeAccel). Such DNAm age AgeAccel biomarkers provide epigenetic age estimates that are independent of chronological age: a positive AgeAccel value indicates that the epigenetic age is higher than expected, whereas a negative value indicates a lower than expected epigenetic age relative to chronological age [41]. With Horvath’s multi-tissue clock [41] this approach revealed significant AgeAccel (P = 0.028) for women with a childhood in Bangladesh, suggesting an accelerated rate of epigenetic ageing [AgeAccel mean: +1.5 (Figure 1a)]. In contrast, a childhood in the UK was associated with decreased AgeAccel, implying that these women experience slower rates of epigenetic ageing [AgeAccel mean: −3.2 (Figure 1a)]. No significant differences were observed between the ‘UK’ and the ‘Bangladeshi’ groups with AgeAccel biomarkers derived from the ‘skin & blood’ clock and the ‘3-CpG-swab’ model (Additional File: Table 2 & Figure S2).

**a)** Plot of DNAm AgeAccel residuals derived from Horvath’s multi tissue clock, where each data point represents a value from an individual. Colours identify the corresponding dataset: blue = childhood in UK (n = 11), orange = childhood in Bangladesh (n = 14). The median is indicated by a black horizontal line. A positive or negative AgeAccel value indicates that the estimated epigenetic/biological age of the sample is higher or lower, respectively, than expected. **b)** The estimated fraction of epithelial cells in buccal samples based on a 2-CpG buccal cell signature model [40]. Asterisks denote two-tailed significance (* = P ≤ 0.05; ** P ≤ 0.01) between groups, based on unpaired t-tests.

Our finding of epigenetic age differences based on Horvath’s multi-tissue clock between women associated with two different geographic childhood environments rests on a small number of sampled individuals. We interpret this result with caution and our finding will be strengthened by independent validation and larger datasets.

The composite nature of buccal tissue could, in part, explain some of the variation in our AgeAccel measurements. In fact, single-cell RNA sequencing revealed the presence of at least eight different subtypes of cells in the buccal mucosal basal layer; turnover of these cells follows a pattern of neutral drift dynamics and is influenced by age and environmental factors [44]. The range of individual DNAm age estimates from buccal epithelial cells is indeed broader, when compared with intra-individual DNAm age estimates from blood-cells, as Hannon and colleagues found, when they assessed Horvath’s epigenetic clock across tissues and cells [45]. Nevertheless, the same study showed that the mean of these epigenetic age predictions was more accurate for buccal samples than for blood samples [45]. We therefore considered the composition and proportion of cells in our buccal samples. Deconvolution methods for DNA methylation data have been developed that provide estimates of the abundance of certain cell subtypes [46,47]. The tissue predicting tool, integrated with the online methylation age calculator [48], determined ‘buccal/saliva’ as the most likely tissue source for all the DNA samples described here (Additional File: Table 2). Wagner and colleagues developed a model based on methylation levels of two CpG sites, which estimates the proportions of buccal epithelial cells and leukocytes in buccal swab samples [40]. With is ‘buccal-cell-signature’ model, we observed a significant difference (P = 0.006) in epithelial cell composition between the UK and Bangladeshi samples (Figure 1b). The overall lower proportions of epithelial cells estimated to be present in buccal swabs from women with a Bangladeshi childhood cannot be easily attributed to differences in collection and processing. Buccal swabs were taken randomly from individuals with UK and Bangladeshi childhoods, but always under supervision of the same person, ensuring consistency of the collection procedure (see also Additional File: Figure 3).

Altered hormone levels could also have contributed to the variation and differences in AgeAccel between the two cohorts. Reproductive hormone levels such as oestrogen and progesterone appear to influence measures of AgeAccel obtained from buccal epithelium, in contrast to blood-derived AgeAccel estimates [49]. That is, menopausal hormone therapies (MHT) are associated with significantly lower epigenetic AgeAccel in buccal tissue [49]. While it is unlikely that the women in our study, aged between 18 and 35 years, were undergoing MHT, we cannot rule out the possibility that some might have used oral contraceptives at the time of buccal sample collection. Alternatively, we could argue that the childhood environment has led to comparatively higher levels in reproductive hormones in the UK group of women, as we previously reported [36], resulting in decreased epigenetic AgeAccel. Future epigenetic age studies using buccal DNA samples will benefit from integrating additional information about the participants.

Duration, intensity and the type of exposure experienced during early life may influence methylation of some clock-associated CpG sites [50]. At this stage, we know that immunological challenges play a role [30,39], but defining the timeframe of exposures during early and middle childhood will be critical to understand complex phenotypic outcomes that appear to be associated with the tick rate of the epigenetic clock. Accelerated epigenetic ageing is consistent with our previous observations of shorter reproductive lifespans and chronically lower levels of reproductive hormones in women who grew up in Bangladesh [28,36,38] (reviewed in [30]), as well as with reduced oocyte yields at the time of retrieval in women undergoing fertility evaluation [51].

## Epigenetic stability of a clock locus

**Figure 2.**
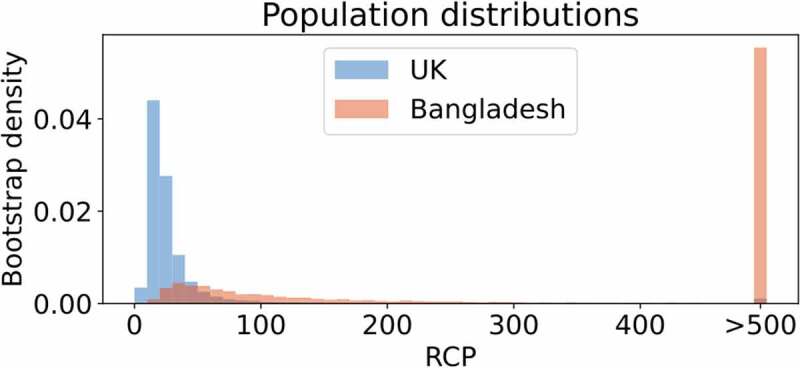
Inferences of DNA methylation stability at the epigenetic-clock associated *LHCGR/LHR* locus.

We used the RCP metric [43] to estimate epigenetic stability in samples, where sufficient DNA was available (Bangladeshi-born, n = 6 / UK-born, n = 6). Specifically, a ~ 100 bp sequence of the *Luteinizing Hormone/Choriogonadotropin Receptor* (*LHCGR/LHR*) locus was analysed, which contains one of the CpG sites comprising Horvath’s multi-tissue epigenetic clock, along with flanking CpG sites (Additional File: Figure 6 and Table 3). This clock-associated CpG site was chosen because *LHCGR/LHR* is necessary for ovulation and luteal formation in females [52,53], and its epigenetic state could be associated with differences in reproductive function, a phenotype we previously linked with altered environmental conditions [28,30,36,54]. We found that RCP estimates were generally higher for the adult migrants who spent their childhoods in Bangladesh (Figure 2). Higher RCP estimates indicate higher levels of epigenetic stability [43]. That is, the methylation states of five or eight CpG dyads (see Additional File: Figure 6), at this *LHCGR*-associated locus are more often identical on the two strands of individual DNA molecules of adult migrants compared with those inferred from DNA samples of ‘second generation’ Bangladeshi women, who grew up in the UK. We note that the RCP estimates are based on a relatively small number of data points (Additional File: Figure 4 and Figure 5), derived from only a single genomic locus. The results are consistent with the idea of what clock-CpGs might proxy: the workings of the epigenetic maintenance system [17]. In our case, subtle differences between the two groups of women is inferred by RCP analysis. Whether variation in the efficiency of the methylation maintenance system is locus-specific, or a genome-wide phenomenon remains to be demonstrated by analysing additional genomic loci or using data obtained from high-resolution deep hairpin bisulphite sequencing (DHBS) [55,56].

The analysis is based on a sampled population of double-stranded DNA molecules and the corresponding methylation states at matching CpG sites (CpG dyads) on the parent and daughter strand of individual molecules. Ratio of Concordance Preference (RCP) is a metric that infers stability/flexibility of methylation states at CpG dyads, without assuming any specific enzymatic mechanisms of DNA methylation. Flexibility, indicated by RCP values near 1, imply that the methylation system has no preference for either concordance or discordance of the methylation state at a CpG dyad and is distributed as expected under a null model, in which methyl groups are placed at random on both strands of a DNA molecule. High RCP values – with the extreme approaching infinity – indicate high stability, where epigenetic maintenance systems have complete preference for concordant methylation states of CpG dyads (they are either methylated or unmethylated); none, or very few CpG dyads are hemi-methylated. Shown are the RCP distributions taken from bootstrap samples, weighing each individual evenly within each group. The sampled population of double-stranded DNA molecules – and the corresponding methylation states of CpG dyads – revealed that the epigenetic maintenance system operates with a tendency of increased stability at the *LHCGR/LHR* locus in women with a childhood in Bangladesh, when compared to the group of women with a childhood in London/UK.

## Conclusions

The results of our study support a large body of work demonstrating phenotypic plasticity in response to environments encountered during early life. A childhood in Bangladesh appears to be associated with a faster rate of epigenetic/biological ageing in young adult females, when compared to women of a similar chronological age (18–35 years) and cultural background, who were born and brought up in London, UK. The multi-tissue epigenetic clock is thought to register the workings of developmental and epigenetic maintenance systems linking these processes across the life course [17,41]. Along with work by Martin-Herranz and colleagues [57], this study is one of the first to explore if differences in function of the epigenetic maintenance system can be linked with epigenetic age estimators. Our RCP measurements detected subtle differences in the stability of epigenetic states, which could be a result of minute changes in the activity of the DNA methylation maintenance system. Alternatively, the RCP measurements may have detected altered DNA methylation stabilities that resulted from differences in the proportions of dividing buccal cell subtypes, leading in sum to dissimilar epigenomes in our two groups of DNA samples.

## Methods

### Collection of buccal cell samples

Buccal cell samples were collected from donors of both, the ‘UK group’ and the ‘Bangladeshi group’ within a ~ two-month period (May–June 2016), all under supervision by KB, thereby ensuring consistency in the process. Swabs were placed in an iSWAB™ collection device containing a proprietary lysis and stabilizing buffer following the instructions of the iSWAB DNA Collection Kit (Mawi DNA Technologies). The lysed buccal cell samples from all individuals were stored under same conditions until further processing.

## DNA methylation data, establishment of DNAm Age and AgeAccel

Genome-wide cytosine methylation levels were established using the Illumina HumanMethylationEPIC BeadChip Array following isolation of genomic DNA from the collected and lysed buccal cell samples (DNeasy Blood & Tissue Kit (Qiagen). Multidimen-sional scaling (MDS) plots indicated that no significant batch effects were skewing the MethylationEPIC BeadChip data sets. The data were processed with the Bioconductor/minfi package. CpG probes associated with known SNPs were removed, as were those with a detection probability of <0.01. Probes on both X and Y chromosomes were retained. Methylation beta values (0–1) were analysed using the Horvath online multi-tissue epigenetic clock [48] and the skin-blood clock [42] following two normalization procedures (SWAN, Quantile) or no-normalization (raw data); for the ‘3-CpG model’[40] only raw (no normalization) data were used. The methylation data set (GSE133355 study) is accessible on the Gene Expression Omnibus (GEO) data platform at:https://www.ncbi.nlm.nih.gov/geo/query/acc.cgi?acc=GSE133355

Files with the beta values obtained from the Illumina HumanMethylationEPIC BeadChip Array work (see above) were used to establish the epigenetic age (DNAm age and AgeAccelerationResiduals – AgeAccel) with Horvath’s method [41]. The underlying algorithms are available through the online DNAm age calculator (https://dnamage.genetics.ucla.edu/new). We used the default setting ‘Normalize Data’ during the online-submission process to this DNAm Age calculator as it often improves the predictive accuracy, since this normalization method renders the input data comparable to the training data of the ‘Horvath clock.’ Output from this online calculator also gives predictions of sex and tissue/cell source of the samples, and this confirmed that all our samples described here were indeed female and the source of tissue was buccal/saliva.

## Generation of bisulphite hairpin data/methylation states of CpG dyads at the *LHCGR/LHR* locus

We have previously described in detail the concept and procedure of generating authenticated, non-redundant double-stranded DNA methylation data [58–60]. In brief, genomic sequence information surrounding the *LHR* clock-CpG site [one of the 353 CpG sites contributing to Horvath’s clock [41]. Illumina cluster ID cg12351433/chr2:48,982,957–48,982,957/UCSC Genome Browser (GRCh37/hg19)] was used to identify suitable restriction recognition sites to generate 3’, or 5’-overhangs, respectively, for the ligation of UMI-barcoded hairpin linkers. Specifically, restriction enzymes StyI or BstXI (New England Biolabs) were used. Combinations of the following primers were used to amplify hairpin-linked, bisulphite converted DNA:

bsLHR-R15’-RCAAATCAAAACAAAACAAACTC-3’;

bsLHR-R25’-CACTAAACACTATCRCAAATCAAAAC-3’;

bsLHR-F15’-TAGTAGGAAGGAGGTTATTGG-3’;

bsLHR-F2 5’-GTAGGTTAAGGTAGAGTAGATTTAG-3’;

bsLHR-F35’-GAATTGGGTTTTTGCGGTTTGTTAG-3’.

Further information of the hairpin-concept and of the barcoded and batch-stamped hairpin linkers (Eurofins Genomics) are provided in Additional File 1.

### Processing of the sequencing data:

Fold is a web application for the analysis of the output of hairpin-bisulphite sequencing data. Specifically, the programme reconstructs, visualizes, and generates statistics on the double-stranded CpG methylation patterns of the original cohort of DNA molecules. This is achieved by first ‘realigning’ the top and bottom strand of the molecule about the hairpin, in which the programme attempts to manage ‘PCR slippage,’ and other sequencing errors. The algorithm then identifies and categorizes CpG dyads, which is possible due to the previous bisulphite conversion of unmethylated cytosine to uracil (and so recognized as tyrosine when sequenced). For example, fully methylated dyads are those regions where the reconstructed top strand is C-G and the bottom is G-C. Similarly, fully unmethylated dyads are those where the top is T-G and the bottom is G-T. In addition, the programme calculates a metric: ‘Ratio of Concordance Preference’ which quantifies the concordance of methylation between the top and bottom strands of the DNA molecule (0 = complete discordance, 1 = random concordance, ∞ = complete concordance). This metric represents the preference of the summation of epigenetic mechanisms of the cell to either maintain or obscure methylation patterns of the DNA in the cells at the time the sample was taken. The functions of Fold were written in R and the web application is written in PHP. The live web application can be found at http://www.gregoryleeman.com/fold, and the repository can be found at https://github.com/gregoryleeman/fold.

## Analysis and comparison of RCPs at the *LHCGR/LHR* locus

RCP values are based on double-stranded DNA methylation data derived from sequences of individual hairpin bisulphite PCRs products. RCP is defined as:

$$RCP=\frac{\sqrt{U\left(U+2m-1\right)}}{1-U-m}$$

RCP analyses were undertaken following the procedures described in [43], with the small addition of bootstrapping individuals within each population. The additional step in the procedure helps to address the possibility of uneven sampling from a larger population of individuals. The analysis procedures in brief are described below.

Each population RCP distribution was drawn through hierarchical bootstrap sampling. For each of 20,000 bootstrap samples, individuals in each population were sampled with replacement, and double stranded DNA sequences of each of the sampled individuals were in turn sampled with replacement. Dyad counts were then normalized, such that each individual had the same number of dyads. The normalized dyad counts were then summed, corrected for failed bisulphite conversions (rate of 0.0039, measured empirically) and inappropriate conversions (rate of 0.017, estimated as described in [43] and used to compute the RCP value. A bootstrap sample of the RCP difference was computed by taking the difference of the RCP values sampled for the two populations.

Assuming that there are no locus-specific differences between the two groups, differences in numbers of CpG Dyads should not affect the results of RCP analysis. RCP treats each dyad independently and does not rely on sequential aspects of the dyads in the data. The number of CpG sites per hairpin, however, does influence the variance of the data, as sites on a single strand are correlated. We take this into account in our hierarchical bootstrapping approach.

For one-tailed comparison tests, with which we examine directional differences, we determined the p-value as the proportion of bootstrap-difference samples to the left of 0. For two-tailed tests, with which we can detect differences in any direction, we determined the p-value as twice the smaller proportion of the bootstrap difference samples on either side of 0.

## Supplementary Material

Supplemental Material

## Data Availability

The datasets generated and/or analysed during the current study are available in the Gene Expression Omnibus (GEO) data platform https://www.ncbi.nlm.nih.gov/geo/query/acc.cgi?acc=GSE133355 All data generated or analysed during this study are included in this published article and its supplementary information file.
