# Supplementary material for: Childhood environment influences epigenetic age and methylation concordance of a CpG clock locus in British-Bangladeshi migrants
Source: Epigenetics. 2022 Dec 10;18(1):2153511. doi: 10.1080/15592294.2022.2153511 (PMC9980690; doi:10.1080/15592294.2022.2153511)
Supplement: Supplemental Material [file KEPI_A_2153511_SM4767.docx]

**ADDITIONAL FILE**

**Childhood environment influences epigenetic age and methylation concordance of a CpG clock locus in British-Bangladeshi migrants**

Reinhard Stöger^1*^, Minseung Choi^2^, Khurshida Begum^3^, Gregory Leeman^1^, Richard D. Emes^4,5^, Philippa Melamed^6^, Gillian R. Bentley^5,7^

^1^ School of Biosciences, University of Nottingham, LE12 5RD, United Kingdom

^2^ School of Medicine, Stanford University, USA

^3^ Department of Anthropology, Durham University, Durham, United Kingdom

^4^ School of Veterinary Medicine and Science, University of Nottingham, LE12 5RD, United Kingdom.

^5^ Advanced Data Analysis Centre, University of Nottingham, LE12 5RD, United Kingdom.

^6^ Faculty of Biology, Technion-Israel Institute of Technology, Haifa, Israel

^7^ Wolfson Research Institute for Health and Wellbeing, Durham University, Durham, United Kingdom

**Additional Data and Results**

**1A 1B**

**Fig 1 / Additional file: DNAm age as a function of chronological age**

**1A:** Scatter plot showing the relationship between chronological age (x-axis) and the predicted epigenetic age (DNAm Age) of individuals (y-axis); analysis was carried out using GraphPad Prism version 9.0.0 for Mac, GraphPad Software, San Diego, California USA, [www.graphpad.com](http://www.graphpad.com). DNAm Age values used for this analysis were obtained from Horvath’s multi-tissue (mt) epigenetic clock. The slopes of the regression lines between the ‘UK’ group (blue / n=11) and the ‘Bangladesh’ group (orange / n=14) are not significantly different (F = 1.5; DFn = 1, DFd = 21 p=0.23). Therefore, a single slope for data of the entire cohort can be established (grey line): the pooled slope equals 0.83. The intercepts are significantly different (F = 8.3. DFn = 1, DFd = 22, p=0.009). ‘UK’ = Bangladeshi women who grew up in London, UK (blue); ‘Bangladesh’ = Bangladeshi women who grew up in Sylhet, Bangladesh (orange). Each data point represents information from an individual.

**1B**: Scatter plot showing the relationship between chronological age (x axis) and the predicted epigenetic age (DNAm Age) of individuals (y-axis) after establishing a minimally adequate model for the data conducted by ANCOVA analysis (carried out using GenStat v.21 /VSN International, Hemel Hempstead / see also *Table 1*). The slopes of the regression lines between the ‘UK’ group (blue / n=11) and the ‘Bangladesh’ group (orange / n=15) are not significantly different.

**Table 1 / Additional file**

| **ANCOVA effects of chronological age and country on DNAm age.**  P values in bold indicate significant effects. Both chronological age and country affected DNA age but without significant interaction. | | | | | |
| --- | --- | --- | --- | --- | --- |
| **Source** | **d.f.** | **SS** | **MS** | **F-ratio** | **P** |
| Chronological age | 1 | 309.57 | 309.57 | 9.18 | **0.006** |
| Country | 1 | 282.95 | 282.95 | 8.39 | **0.008** |
| Chronological age × Country interaction | 1 | 38.32 | 38.32 | 1.14 | 0.298 |
| Residual | 22 | 741.94 | 33.72 |  |  |
| Total | 25 | 1723.29 | 68.93 |  |  |

For this ANCOVA analysis one data point (individual) was included, which we later removed from all subsequent analyses described in the manuscript, as it failed to meet a quality control criterion – removal did not affect the results or conclusions. ANCOVA analysis was carried out using GenStat v.21 /VSN International, Hemel Hempstead.

**Table 2 / Additional file**

**Estimates of DNA methylation age (DNAm Age), AgeAccel and tissue composition**

An overview of the different epigenetic age estimates, AgeAccel estimates and tissue/cell type predictions, using results from ‘Horvath’s multi-tissue’ clock ( <https://dnamage.genetics.ucla.edu/new> ), a ‘skin & blood’ clock (Horvath et al. 2018 / DOI: [10.18632/aging.101508](https://doi.org/10.18632/aging.101508) ) and a ‘3-CpG-buccal swab’ model (Eipel et al. 2016 / [DOI:10.18632/aging.100972](https://www.aging-us.com/article/100972) ). The array IDs allow retrieval of the raw Infinium MethylationEPIC BeadChip data on the Gene Expression Omnibus (GEO) data platform at:
<https://www.ncbi.nlm.nih.gov/geo/query/acc.cgi?acc=GSE133355>


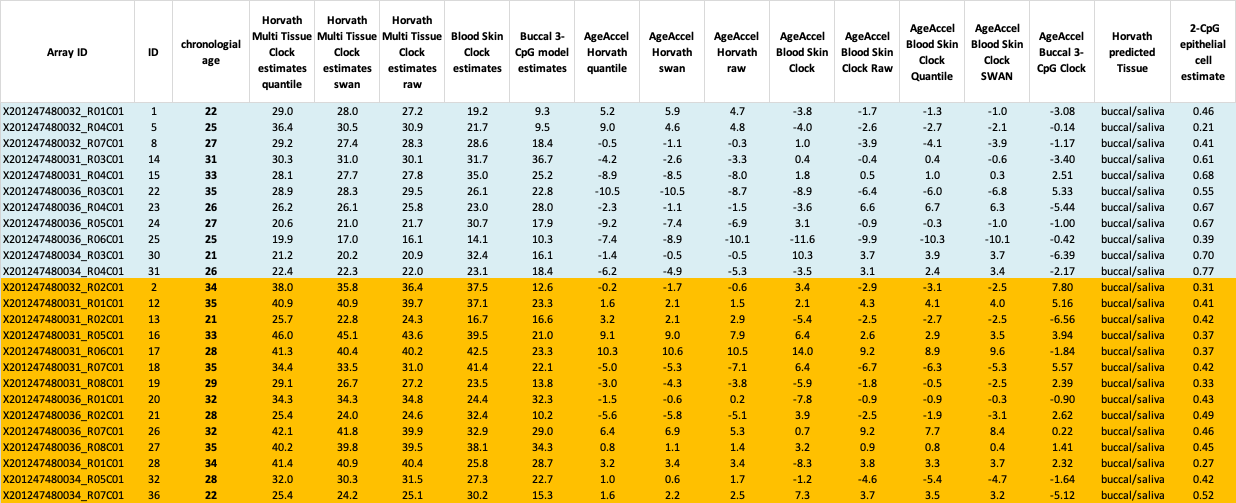

**Fig 2 / Additional file:** Scatter plots showing the distribution of AgeAccel residuals derived from different epigenetic age estimators (grey lines above the scatterplots): ‘Horvath’s multi-tissue’ clock ( Horvath 2013 / <https://doi.org/10.1186/gb-2013-14-10-r115> ), a ‘skin & blood’ clock (Horvath et al. 2018 / [DOI: 10.18632/aging.101508](https://doi.org/10.18632/aging.101508) ) and a ‘3-CpG-swab’ model (Eipel et al. 2016 / [DOI:10.18632/aging.100972](https://doi.org/10.18632/aging.100972) ). DNA methylation beta values can be normalised using several methods; ‘raw’ beta values (no-normalisation) or SWAN or QUANTILE normalised beta values, respectively were used here to generate epigenetic age estimates and subsequently AgeAccel residuals (*see also table 2 / additional file*). Different normalisation methods did not generate markedly different results compared to the outputs generated by ‘raw’ beta values.

**Fig 3 / Additional file: epithelial cell fraction - AgeAccel**

Scatter plot showing the relationship between the estimated fraction of epithelial cells in buccal samples and AgeAccel. Estimates of epithelial cell fractions were obtained using the 2-CpG ‘buccal signature model’ established by Eipel and colleagues ( 2016 / [DOI:10.18632/aging.100972](https://www.aging-us.com/article/100972) ). Age Accel residuals were derived from epigenetic age predictions using the ‘3-CpG buccal estimator (Eipel et al. ( 2016 / [DOI:10.18632/aging.100972](https://www.aging-us.com/article/100972) ). Each data point represents an individual data pair (fraction epithelial cell/corresponding Age Accel estimate. The difference between the slopes is not significant: (F = 0.1271. DFn = 1, DFd = 21, P=0.73). That is, the fraction of epithelial cells has no significant influence on Age Accel. Blue/UK slope: -0.013; orange/Bangladesh slope: -0.008. Blue dots represent individuals from the ‘UK group’ = Bangladeshi women who grew up in London, UK; orange dots: ‘Bangladesh group’ = Bangladeshi women who grew up in Sylhet, Bangladesh.

**Guide to interpret the double-stranded DNA methylation data show in Fig 4 / additional file (below)**

The hairpin bisulfite sequencing data (Fig 4 / Additional file) are processed in that the methylation status of matching cytosines in CpG sites of the top- and bottom strands (CpG dyads) of individual, double-stranded DNA molecules are indicated as methylated (=1), or unmethylated (=0). Hemi-methylated sites: the top strand is methylated, whereas the bottom strand is unmethylated, or vice versa (=1/0 or 0/1); examples for hemi-methylated sites are data from molecule 5_5 and 20_9 (see below)

**Additional Fig 4**

**Hairpin methylation data from individuals with a Bangladesh-childhood**

12_1 0,0,0,0,0,0,1,0,0 GTTAAATT

0,0,0,0,0,0,1,0,0

12_2 0,0,0,0,0,0,0,0,1 GTATTATT

0,0,0,0,0,0,0,0,1

12_3 0,0,0,0,0,0,0,0,1 TGGTTTTA

0,0,0,0,0,0,0,0,1

12_4 0,0,0,0,0,0,0,0,0 GGTTGAGG

0,0,0,0,0,0,0,0,0

12_5 0,0,0,0,0,0,0,0,0 ATTTGTTG

0,0,0,0,0,0,0,0,0

12_6 0,0,0,0,0,0,0,0,0 TAGGAGTG

0,0,0,0,0,0,0,0,0

12_7 0,0,0,1,0,0,0,0,0 GGTTTGAA

0,0,0,0,0,0,0,0,0

12_8 0,0,0,0,0,0,0,0,0 GGTGTGTT

0,0,0,0,0,0,0,0,0

12_9 0,0,0,0,0,0,1,0,0 AGATGTAA

0,0,0,0,0,0,1,0,0

12_10 0,0,0,1,0,0,0,0,0 TTGGAGGG

0,0,0,1,0,0,0,0,0

12_11 0,0,0,0,0,0,0,0,0 ATTTAGTT

0,0,0,0,0,0,0,0,0

12_12 0,0,0,0,0,0,0,0,0 TGTAAAGA

0,0,0,0,0,0,0,0,0

12_13 0,0,1,1,0,0,0,0,0 TGTTTTTT

0,1,1,1,1,0,0,0,0

12_14 0,0,0,0,0,0,0,0,0 ATTTTAGG

0,0,0,0,0,0,0,1,0

12_15 0,0,0,0,0,0,0,0,0 TTGGATTT

0,0,0,0,0,0,0,0,0

12_16 0,0,0,0,0,0,0,0,0 TTGAAAGG

0,0,0,0,0,0,0,0,0

12_17 0,0,0,0,0,0,0,0,0 GGGTTAAG

0,0,0,0,0,0,0,0,0

13_1 0,0,0,0,1,1,0,0,0 AAGAGAGT

0,0,0,0,0,0,0,0,0

13_2 0,0,0,0,0,0,0,0,0 TTGTTATA

0,0,0,0,0,0,0,0,0

13_3 0,0,0,0,0,0,0,0,0 TAAATGTA

0,0,0,0,0,0,0,0,0

13_4 0,0,0,1,0,0,0,0,1 TTAGATGG

0,0,0,1,0,0,0,0,1

13_5 0,0,0,0,0,0,0,0,0 TGATATAA

0,0,0,0,0,0,0,0,0

13_6 0,0,0,0,0,0,0,1,0 GGGTTGGG

0,0,0,0,0,0,0,0,0

13_7 0,0,0,0,0,0,0,0,0 GATTGATA

0,0,0,0,0,0,0,0,0

13_8 0,0,0,0,0,0,0,0,0 GGGTTAAG

0,0,0,0,0,0,0,0,0

13_9 0,0,0,0,0,0,0,0,0 TAGTATAT

0,0,0,0,0,0,0,0,0

13_10 0,0,0,0,0,0,0,0,0 AAAAATAA

0,0,0,0,0,0,0,0,0

13_11 0,0,0,0,0,0,0,0,0 ATAGGAAG

0,0,0,0,0,0,0,0,0

13_12 0,0,0,0,0,0,0,0,1 TGGGATGA

0,0,0,0,0,0,0,0,1

13_13 0,0,0,0,0,0,0,0,0 TATGTTTG

0,0,0,0,0,0,0,0,0

13_14 0,0,0,0,1,1,0,0,0 GAGAAATG

0,0,0,0,1,1,0,0,0

13_15 0,1,0,0,0,0,0,0,0 TTTATTGT

0,1,0,0,0,0,0,0,0

13_16 0,0,0,0,0,0,0,0,0 TTTGTAGA

0,0,0,0,0,0,0,0,0

13_17 0,0,0,0,0,0,0,0,0 ATGTTAGT

0,0,0,0,0,0,0,0,0

13_18 0,0,0,0,0,0,0,0,0 ATGAGGGG

0,0,0,0,0,0,0,0,0

13_19 0,0,0,0,0,0,1,0,0 GTTTGAAG

0,0,0,0,0,0,0,0,0

19_1 0,0,0,0,0,0,0,0,0 GTGAATAT

0,1,0,0,0,0,0,0,0

19_2 0,0,0,0,0,0,0,0,0 TAGGGAAT

0,0,0,0,0,0,0,0,0

19_3 0,0,0,0,0,0,1,1,1 GTGGGGTG

0,0,0,0,0,0,1,1,1

19_4 0,0,0,0,0,0,0,0,0 GAAATAGT

0,0,0,0,0,0,0,0,0

19_5 0,0,0,0,0,0,1,0,1 GTGTGTAT

0,0,0,0,0,0,1,0,1

19_6 0,0,0,0,0,0,0,0,0 TGATGTGA

0,0,0,0,0,0,0,0,0

19_7 0,0,0,0,0,0,0,0,0 ATAGTAAT

0,0,0,0,0,0,0,0,0

19_8 0,0,0,0,0,0,0,0,0 ATATGTTC

0,0,0,0,0,0,0,0,0

19_9 1,1,0,0,0,0,0,0,0 AGTTGAGG

1,1,0,0,0,0,0,0,0

19_10 0,0,0,0,0,0,0,0,0 TTTAAAAA

0,0,0,0,0,0,0,0,0

19_11 0,0,0,0,0,0,0,0,0 GATAATTT

0,0,0,0,0,0,0,0,0

19_12 0,0,0,0,0,0,0,0,0 GTTGTTGT

0,0,0,0,0,0,0,0,0

19_13 0,0,0,0,0,0,0,0,0 TTGTGTAT

0,0,0,0,0,0,0,0,0

19_14 0,1,1,0,0,0,0,0,0 TTGGGTAT

0,1,1,0,0,0,0,0,0

19_15 0,0,0,0,0,0,1,0,0 ATTTATTT

0,0,0,0,0,0,1,0,0

19_16 0,0,0,1,0,0,0,0,0 GTGGAGGT

0,0,0,1,0,0,0,0,0

19_17 0,0,0,0,0,0,0,0,1 TAAGTAGG

0,0,0,0,0,0,0,0,1

19_18 0,0,0,0,0,1,0,0,0 AATGTTGT

0,0,0,0,0,1,0,0,0

19_19 0,0,0,0,0,0,0,0,0 TTAAGGGT

0,0,0,0,0,0,0,0,0

19_20 0,0,0,0,0,0,0,0,0 TTAAAGGT

0,0,0,0,0,0,0,0,0

19_21 0,0,0,0,0,0,0,0,0 TAGATTTG

0,0,0,0,0,0,0,0,0

19_22 0,0,0,0,0,0,0,0,0 GGGATTTT

0,0,0,0,0,0,0,0,0

19_23 0,0,0,0,0,0,0,0,0 AGATTGTG

0,0,0,0,0,0,0,0,0

19_24 0,0,0,0,0,0,0,0,0 TTAATAAT

0,0,0,0,0,0,0,0,0

20_1 0,0,1,1,1 AGAGATGG

0,0,1,1,1

20_2 0,0,1,1,1 GAAGGATA

0,0,1,1,1

20_3 0,0,1,1,1 TAGGTAGG

0,0,1,1,1

20_4 0,0,0,0,0 GGGGATGG

0,0,0,0,0

20_5 0,0,0,0,0 AAATGTGG

0,0,0,0,0

20_6 0,0,0,0,0 GTAGTTTA

0,0,0,0,0

20_7 0,0,1,0,1 ATAAAGAG

0,0,1,0,1

20_8 1,1,1,0,1 GTGAGGAG

1,1,1,0,1

20_9 0,0,0,0,0 GATGGGGT

0,0,1,0,1

20_10 0,0,0,0,0 TATGAGTG

0,0,0,0,0

20_11 0,0,0,0,0 AAAAATTG

0,0,0,0,0

21_1 1,1,1,1,1 ATGAGGAT

1,1,1,1,1

21_2 1,1,1,1,1 GGTGATTG

1,1,1,1,1

21_3 1,1,1,1,1 TGGGATAA

1,1,1,1,1

21_4 1,1,1,1,1 GGATGTGA

1,1,1,1,1

21_5 1,1,1,1,1 TAAGGGGG

1,1,1,1,1

36_1 0,1,0,1,0 GGAGAAGT

0,1,0,1,0

36_2 0,0,0,0,0 AGGGAGTT

0,0,0,0,0

36_3 0,0,0,0,0 GGAGAGGA

0,0,0,0,0

36_4 0,0,0,0,0 GGAGAGGG

0,0,0,0,0

36_5 0,0,0,0,0 AGAGTAGT

0,0,0,0,0

36_6 0,0,0,0,0 AGGGAGGA

0,0,0,0,0

36_7 0,0,0,0,0 AAATTTAG

0,0,0,0,0

36_8 0,0,0,0,0 GGGTAATA

0,0,0,0,0

36_9 0,0,0,0,0 ATATGGAG

0,0,0,0,0

36_10 0,0,0,0,0 AAGAAAAA

0,0,0,0,0

36_11 0,0,0,0,0 ATAAGAAA

0,0,0,0,0

36_12 0,0,0,0,1 AAGGGGTA

0,0,0,0,1

**Hairpin methylation data from individuals with a UK-childhood**

5_1 0,0,0,0,0 AGTTGTTT

0,0,0,0,0

5_2 0,0,1,0,0 GGATGATA

0,0,1,0,0

5_3 1,1,1,1,1 GATAGTTG

1,1,1,1,1

5_4 1,1,1,1,1 GTTTAGGT

1,1,1,0,1

5_5 0,0,0,0,1 TGGGGTGA

0,0,0,0,0

15_1 0,0,0,0,0,0,0,0,0 ATATGAGA

0,0,0,0,0,0,0,0,0

15_2 0,0,0,0,0,0,0,0,0 ATGGGAGA

0,0,0,0,0,0,0,0,0

15_3 0,0,0,0,0,0,0,0,0 GATTGTAA

0,0,0,0,0,0,0,0,0

15_4 0,0,0,0,0,0,1,0,1 ATAGGGAA

0,0,0,0,0,1,1,0,1

15_5 0,0,0,0,0,0,1,0,0 TTGAAGAT

0,0,0,0,0,0,1,0,1

15_6 0,0,0,0,0,0,0,0,0 TGGTTAAA

0,0,0,0,0,0,0,0,0

15_7 0,0,0,0,0,0,0,0,0 TAAAGAAA

0,0,0,0,0,0,0,0,0

15_8 0,0,0,0,0,0,0,0,0 TATTTTTT

0,0,0,0,0,0,0,0,0

15_9 0,0,0,0,0,0,0,0,0 TGTATGTA

0,0,0,0,0,0,0,0,0

15_10 0,0,1,0,0,0,0,0,0 AAGTAAAG

0,0,0,0,0,0,0,0,0

15_11 1,1,0,0,0,0,1,0,0 AAAATTGA

1,1,0,0,0,0,1,0,1

15_12 0,0,0,0,0,0,0,0,0 GTGTGGGG

0,0,0,0,0,0,0,0,0

15_13 0,1,0,0,0,0,0,0,0 TAGTTGGA

0,1,0,0,0,0,0,0,0

15_14 1,1,1,1,1,0,0,0,1 AATGTAAA

1,1,1,1,0,0,0,0,1

15_15 0,0,0,0,0,0,0,0,0 TTGGTAGG

0,0,0,0,0,0,0,0,0

15_16 0,0,0,0,1,0,0,0,1 GTATTATG

0,0,0,0,0,0,1,0,1

30_1 0,0,0,0,0,0,0,0,0 ATAAGGGA

0,0,0,0,0,0,0,0,0

30_2 0,0,0,0,0,0,0,0,1 TTTTTAGA

0,0,0,0,0,0,0,0,0

30_3 0,0,0,0,0,0,0,0,1 TTGTGATA

0,0,0,0,0,0,0,0,1

30_4 0,0,0,0,0,0,0,0,0 TATAATTA

0,0,0,0,0,0,0,0,0

30_5 0,0,0,0,1,0,0,0,0 GAGTAATT

0,0,0,0,1,0,0,0,0

30_6 0,0,0,0,0,0,0,0,0 TGAGTGAG

0,0,0,0,0,0,0,0,0

30_7 0,0,0,0,1,0,0,0,0 GGTGAATG

0,0,0,0,0,0,0,0,0

30_8 0,0,0,0,0,0,0,0,0 TGGAAATA

0,0,0,0,0,0,0,0,0

30_9 0,0,0,0,0,0,0,0,0 TTTTTGGA

0,0,0,0,0,0,0,0,0

30_10 0,0,0,0,0,0,1,0,1 GGTAAGTA

0,0,0,0,1,0,1,0,1

30_11 0,0,0,0,0,0,1,0,1 GATAAAAG

0,0,0,0,0,0,0,0,1

30_12 0,0,0,1,1,0,0,0,0 TTAATAGG

0,1,0,1,0,0,0,1,0

30_13 0,0,0,0,0,0,0,0,0 AAGGTTTG

0,0,0,0,0,0,0,0,0

30_14 0,0,0,0,0,0,0,0,0 AGTTGGAG

0,0,0,0,0,0,0,0,0

30_15 0,0,0,0,0,0,0,0,0 TAAAGTAG

0,0,0,0,0,0,0,0,0

30_16 0,0,0,0,0,0,1,0,1 TTTTTAGA

0,0,0,0,0,0,0,0,0

30_17 0,0,0,0,0,0,0,0,0 TTAATTGA

0,0,0,0,0,0,0,0,0

30_18 0,0,0,0,0,0,0,0,0 TGGTTAAT

0,0,0,0,0,0,0,0,0

30_19 0,0,0,1,0,0,0,0,0 ATGTTGTG

0,0,0,0,0,0,0,0,0

30_20 0,0,0,0,0,0,0,0,0 TGAAAGAT

0,0,0,0,0,0,0,0,0

30_21 0,0,0,0,0,0,0,0,0 AGTAAGTG

0,0,0,0,0,0,0,0,0

30_22 0,0,0,0,0,0,0,0,0 TTTTTAGA

0,0,0,0,0,0,0,0,0

30_23 0,0,0,0,0,0,0,0,1 AAATGGGT

0,0,0,0,0,0,0,0,0

31_1 0,1,0,0,0,1,0,0,0 GGATTAGA

0,1,0,0,0,1,0,0,0

31_2 0,0,0,0,0,1,1,1,1 TGAGTAAA

0,0,0,0,0,1,1,1,1

31_3 0,0,0,1,0,0,1,1,1 TAGAGAGA

0,0,0,1,0,0,1,1,1

31_4 0,0,0,0,0,0,0,0,0 TTTATAAT

0,0,0,0,0,0,0,0,0

31_5 1,1,0,0,0,0,1,0,0 AGATATAG

1,1,0,0,0,0,1,0,0

31_6 0,0,0,0,0,0,0,0,0 AAATAAAA

0,0,0,0,0,0,0,0,0

6_1 0,0,0,0,0 ATTGTTGA

0,0,0,0,0

6_2 0,0,1,0,0 TTGGGAAT

0,0,0,0,0

6_3 0,0,0,0,0 TTAAGATG

0,0,0,0,0

6_4 0,1,0,1,1 GTAGGGGG

0,1,0,1,1

6_5 0,0,0,0,0 GGTAGGAT

0,0,0,0,0

8_1 0,0,1,1,0 TATTGGAG

0,0,1,1,0

8_2 0,0,0,0,0 GTTTGTAT

0,0,0,0,0

8_3 0,0,0,0,0 GTGTGTGT

0,0,0,0,0

8_4 0,0,0,0,0 TAGGGGAT

0,0,0,0,0

8_5 0,0,1,0,1 TATTAAAG

0,0,1,0,1

8_6 0,0,0,0,0 GTAGGTGG

0,0,0,1,0

8_7 1,0,1,1,1 GTAGAGGG

1,0,1,1,1

8_8 0,0,0,0,0 AAAATATA

0,0,0,0,0

8_9 0,0,0,0,0 GTTTTTGT

0,0,0,0,0

8_10 0,0,0,0,0 GAGGTTAT

0,0,0,0,1

8_11 0,0,0,0,0 TGTAGGAA

0,0,0,0,0

8_12 0,0,1,1,1 AATTTGTT

0,0,1,1,1

8_13 0,0,0,0,0 AAGTTTAT

0,0,0,0,0

8_14 0,0,0,0,0 TGGATTTG

0,0,0,0,0

8_15 0,0,0,0,0 TATTAGGT

0,0,1,1,1

8_16 0,0,0,0,0 TATTGGAT

0,0,0,0,0

8_17 0,0,0,0,0 AATTGGGT

0,0,0,0,0

8_18 0,0,0,0,0 TGTTGTAA

0,0,0,0,0

8_19 0,0,1,0,1 AGGATATA

0,0,1,0,1

8_20 0,0,1,0,0 AGTTAGGT

0,0,1,0,0

8_21 0,0,0,0,0 TTAGTAAG

0,0,0,0,0

8_22 0,0,1,1,1 GAATTTGT

0,0,1,1,1

8_23 0,0,0,0,0 GAAATGGA

0,0,0,0,0

8_24 0,0,0,0,0 GTTGTTAA

0,0,0,0,0

8_25 1,0,1,1,1 TTTTGGGT

0,0,0,0,0

8_26 0,0,0,0,0 GATAAGGG

0,0,0,0,0

8_27 0,0,0,0,0 GGAAGTTG

0,0,0,0,0

8_28 0,1,1,1,1 AGTAATGT

0,1,1,1,1

8_29 0,0,0,0,0 AAATGGGT

0,0,0,0,0

8_30 0,0,0,0,0 AGTTTGGT

0,0,0,0,0

8_31 0,0,0,0,0 GAGATTTT

0,0,0,0,0

**Fig 4 / Additional file: double-stranded DNA methylation data**

The cytosine methylation status (both top and bottom strand = CpG dyad) of the CpG site contributing to Horvath’s multi-tissue clock is indicated (aqua blue). Cytosine methylation information of flanking CpGs are also shown: depending on the StyI-hairpin linker approach (four CpG dyads), or the BstXI-hairpin linker approach (eight CpG dyads). The different number of sampled CpGs flanking the ‘clock CpG’ was considered and accounted for in the subsequent RCP analyses, leading to a boot-strap approach of pooled samples as described in the paper and below (Figure 5 / Additional file).


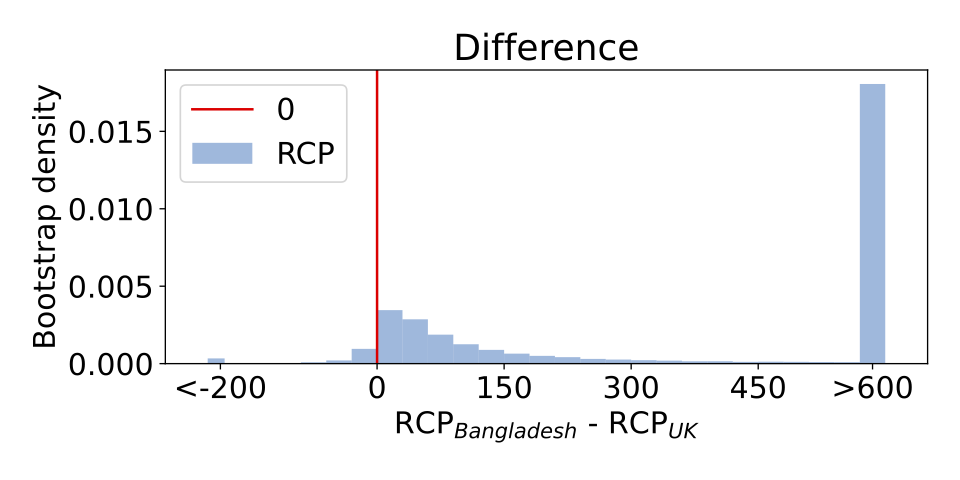


**Fig 5 / Additional file: Nested bootstrapping**

A nested bootstrapping approach to test the significance of the difference in RCP values between the two groups of women (Bangladesh vs UK childhood). Each population RCP distribution was drawn through hierarchical bootstrap sampling. For each of 20,000 bootstrap samples, individuals in each population were sampled with replacement, and double stranded DNA sequences of each of the sampled individuals were in turn sampled with replacement. Dyad counts were normalized, such that each individual had the same number of dyads. Normalised dyad counts were then used to compute the RCP value. A bootstrap sample of the RCP difference was computed by taking the difference of the RCP values sampled for the two populations.

The red line is set at 0. The p-value is derived from this as the proportion of samples to the left of 0. (a one-tailed test to examine whether Bangladesh RCPs are significantly greater than UK RCP values). Two-tailed p-value is the double of that amount: p= 0.026 (one tailed); p= 0.052 (two tailed)

**Additional Methods**

**Fig 6 / Additional file**

**Fig 6 / Additional file: Design of hairpin bisulfite PCR approaches to analyse the *LHCGR/LHR* genomic locus containing a ‘clock CpG’ site**

The human *Luteinizing Hormone/Choriogonadotropin Receptor (LHCGR/LHR)* locus harbours a CpG site [clock CpG] which contributes to Horvath’s multi tissue epigenetic clock ( Horvath 2013 / <https://doi.org/10.1186/gb-2013-14-10-r115> ). This ‘clock CpG site was PCR-amplified following hairpin linker-ligation and sodium bisulfite conversion, along with flanking CpGs (highlighted in red): four CpGs with the StyI-hairpin linker approach and eight CpGs with the BstXI-hairpin linker approach, respectively. Two different DNA restriction enzymes were chosen (StyI restriction site / pink; BstXI restriction site / aqua blue), because PCR efficiency was improved by switching from StyI to BstXI. Limited amount of DNA samples did not allow repeat sampling; the different number of sampled CpGs flanking the ‘clock CpG’ was considered and accounted for in the subsequent RCP analyses, leading to a boot-strap approach of pooled samples as described in the paper and below.

**Table 3 / Additional file**

**BstXI- and StyI-hairpin linkers used in this study**

For each DNA sample (DNA from an individual) a unique “barcode” and “batchstamp” sequence was used to synthesise the hairpin linker. Labelling each genomic template with a “sample ID” and unique molecular identifier allows generation of robust data sets that are free of redundant and contaminant PCR products; we previously described the development and principles of this unique molecular identification system in Miner et al. 2004 / [doi: 10.1093/nar/gnh132](https://doi.org/10.1093%2Fnar%2Fgnh132). Small blue letters indicate the sequences complementary to the ‘sticky ends’ generated by the staggered cut of the restriction enzymes. Red ‘D’s is the degenerate base symbol for the bases A,G,T, which are randomly integrated.
